# Accuracy of 1,2-o-Dilauryl-rac-glycero-3-glutaric Acid-(6′-methylresorufin) Ester (DGGR)-Lipase to Predict Canine Pancreas-Specific Lipase (cPL) and Diagnostic Accuracy of Both Tests for Canine Acute Pancreatitis

**DOI:** 10.3390/vetsci9040177

**Published:** 2022-04-08

**Authors:** Lina A. Wolfer, Judith Howard, Laureen M. Peters

**Affiliations:** 1 Clinical Diagnostic Laboratory, Department of Clinical Veterinary Medicine, Vetsuisse Faculty, University of Bern, 3012 Bern, Switzerland; lina.wolfer@vetsuisse.unibe.ch (L.A.W.); judith.howard@vetsuisse.unibe.ch (J.H.); 2 Division of Small Animal Internal Medicine, Department of Clinical Veterinary Medicine, Vetsuisse Faculty, University of Bern, 3012 Bern, Switzerland

**Keywords:** diagnostic, dog, pancreas

## Abstract

Different lipase assays have variable reported diagnostic accuracies for acute pancreatitis (AP) in dogs. The aims of this retrospective study were to evaluate optimal cutoffs for 1,2-o-dilauryl-rac-glycero-3-glutaric acid-(6′-methylresorufin) ester (DGGR)-lipase to predict diagnostic cutoffs of canine pancreas-specific lipase (cPL; IDEXX). DGGR-lipase activity and cPL from the same blood draw in 301 dogs with a variety of diseases were compared using Spearman’s rank correlation, Cohen’s kappa agreement, and receiver operating characteristic (ROC) curves. Activity of DGGR-lipase (10–15,616 U/L) and cPL concentrations (8.1–≥2000 µg/L) were highly correlated (r_s_ = 0.91). Areas under the ROC curves (AUCs) to predict cPL >200 and ≥400 µg/L with DGGR-lipase were 0.97 and 0.99, with optimal cutoffs of 143 U/L (sensitivity (Se) 91.7%; specificity (Sp) 95.3%) and 205 U/L (Se 97.5%; Sp 96.4%), and Cohen’s kappa agreements of 0.87 and 0.92, respectively. AUCs for a clinical diagnosis of AP, assigned to 87/301 dogs, with DGGR-lipase (0.75) and cPL (0.76) did not differ significantly (*p* = 0.48); optimal cutoffs were 161 U/L for DGGR (Se 67%; Sp 81%) and 235 µg/L for cPL (Se 68%; Sp 84%). To conclude, DGGR-lipase is a highly accurate predictor of cPL with a comparable performance when used to diagnose AP in dogs.

## 1. Introduction

Acute pancreatitis (AP) is a common disease in dogs, generally associated with systemic inflammatory response syndrome and, in severe cases, with disseminated intravascular coagulation, acute renal failure, multiple organ dysfunction syndrome, and death [1,2]. The aetiology and pathophysiology of AP in dogs are not yet fully understood and have been mainly inferred from human medicine and rodent models; suspected underlying causes include dietary factors, hyperlipidaemia, endocrinopathies such as diabetes mellitus, ischaemia, and drug reactions [1,3]. Premature inappropriate activation of proteases such as trypsinogen to trypsin within pancreatic tissue is currently accepted as a key event, triggering autodigestion, necrosis, and severe inflammation [1,3]. Histologically, AP is defined by the presence of neutrophilic inflammation, necrosis, and oedema [3].

The diagnosis of AP is challenging because it relies on a combination of suggestive clinical signs, diagnostic imaging, and elevated serum pancreatic enzymes, all of which have limited diagnostic accuracy. Clinical signs associated with moderate to severe AP are nonspecific and include vomiting, anorexia, abdominal pain, diarrhoea, and hypovolemia, but mild AP may be subclinical. Ultrasound is widely used in small animal practice to assess the pancreas but has limited sensitivity (43–89%) and specificity (43–92%) for AP, which depend on disease severity, operator skills, equipment, and patient-related factors, such as obesity and abdominal pain [4].

Pancreatic enzymes are frequently used as biochemical markers of AP; however, the diagnostic accuracy of the catalytic 1,2-diglyceride lipase (1,2-DiG) and amylase assays are poor to moderate, with sensitivities reported between 43.4–71% and 7–56%, respectively, depending on the severity of the pancreatitis [5,6]. Their specificity varies from 43–92.5% for 1,2-DiG and 76.7–100% for amylase, which has been attributed in part to nonpancreatic sources of enzymes, including the gastric mucosa and liver [5,6], and decreased renal clearance in kidney disease [7,8]. Therefore, pancreatic enzyme increases above threefold the upper reference limit (URL) have been used in the diagnosis of AP in dogs [9]. A newer catalytic lipase activity assay, using the 1,2-o-dilauryl-rac-glycero-3-glutaric acid-(6′-methylresorufin) ester (DGGR) substrate, was found to have superior sensitivity (73.3–93.3%) but similar specificity (53.3–66.6%) compared to the previously used 1,2-DiG assay [10], and is currently in use in several diagnostic laboratories, including our institution. Currently, an immunologic assay, measuring canine pancreas-specific lipase (cPL) immunoreactivity (Spec cPL^®^, IDEXX Laboratories, Westbrook, ME, USA) is considered the test of choice because it detects lipase exclusively of pancreatic origin [2,11]. Nonetheless, increased cPL concentrations have been reported in a variety of nonpancreatic disorders, including septic peritonitis, gastrointestinal foreign bodies, parvoviral enteritis, gastric dilatation–volvulus, cardiac disease, ehrlichiosis, hyperadrenocorticism, glucocorticoid administration, diabetic ketoacidosis, and obesity [12,13,14,15,16,17,18,19,20]. Furthermore, its sensitivity declines from around 70% in dogs with moderate to severe AP to around 20% in those with mild AP [6,12]. Further disadvantages include the relatively high cost and delayed turnaround time, as cPL requires submission to an external laboratory. A point-of-care semiquantitative test, the SNAP cPL^®^ (IDEXX Laboratories, Westbrook, ME, USA), was developed for rapid patient-side diagnosis of pancreatitis but, despite a good sensitivity of 73.9–100%, its specificity lies between 59–77.8% [12,21], and clinicians are encouraged to follow up a positive SNAP cPL^®^ test result with a measurement of cPL concentration [22].

Previous studies demonstrated agreements varying from good to near perfect between DGGR-lipase activity and cPL concentrations in dogs using different cutoffs for DGGR-lipase, but optimal cutoffs to predict cPL were not evaluated [23,24,25]. Additionally, DGGR-lipase activity greater than 3× URL had 80% sensitivity and 100% specificity for cPL concentrations considered consistent with AP in critically ill dogs with a variety of primary clinical diseases, including AP, as well as renal disease, immune-mediated and endocrine disorders, and upper airway obstruction, suggesting the limited specificity of both assays [26].

The aims of this study were to evaluate optimal cutoffs for DGGR-lipase activity to predict cPL, assess the agreement between both tests in dogs with a variety of underlying diseases, and evaluate the diagnostic accuracy of both tests for a clinical diagnosis of AP in dogs. We found that DGGR-lipase activity is a highly accurate predictor of cPL concentration and that both tests have similarly modest diagnostic accuracy for a clinical diagnosis of AP.

## 2. Materials and Methods

A retrospective search of the electronic medical and laboratory databases was performed to identify client-owned dogs presented to the Small Animal Clinic, Vetsuisse Faculty, University of Berne, Switzerland, between April 2015 and April 2021, that had cPL concentrations measured as part of their routine medical workup. All dogs for which measurement of DGGR-lipase activity was available from the same blood draw as cPL concentrations were included, regardless of the reason for presentation. Additionally, cPL concentrations were measured from leftover serum samples, taken as part of routine diagnostic testing from our hospital population, in cases where a DGGR-lipase activity assay was performed but for which cPL concentrations were not initially requested. For these samples, signed owner consent for the use of leftover biological material was available. These serum samples were conserved at −20 °C for up to 5 months and at −80 °C for a maximum of 15 months. Data collected included signalment, clinical diagnosis, ultrasonographic evaluation of the pancreas, cPL concentrations, and DGGR-lipase activities. For dogs with more than one sample submission for cPL concentrations, only the first analysis was included.

Measurements of DGGR-lipase activity were performed at the Clinical Diagnostic Laboratory of the Vetsuisse Faculty Berne, Switzerland, on a clinical chemistry analyser (Cobas c501, Roche Diagnostics, Basel, Switzerland) using an enzymatic assay (LIPC, Ref. 05401704, Roche Diagnostics, Basel, Switzerland) based on the previously validated DGGR-substrate [10,24] according to the manufacturer’s instructions, using either plasma or serum, depending on availability. Our de novo in-house calculated reference interval for DGGR-lipase activity ranges from 25–180 U/L. Serum samples for cPL concentrations were submitted to IDEXX Diavet, Bäch, Switzerland, by overnight courier. According to IDEXX Laboratories, a cPL concentration of ≥400 µg/L is consistent with pancreatitis, whereas concentrations of 201–399 µg/L are considered equivocal, and those ≤200 µg/L inconsistent with pancreatitis.

Clinical diagnoses of AP were made by a board-certified internal medicine specialist or a junior clinician under their direct supervision and were generally based on a combination of clinical signs, including vomiting, anorexia, diarrhea, and abdominal pain, consistent lipase assays (DGGR-lipase activity and/or cPL concentrations), and ultrasound assessment performed by a board-certified radiologist or a diagnostic imaging resident under their direct supervision. In the case of comorbidities, the disease deemed as clinically most significant at the time of the cPL measurement was considered the primary diagnosis.

Data analysis was performed using commercial software (MedCalc^®^ Statistical Software version 20.009, MedCalc Software Ltd., Ostend, Belgium; 2021). Normality was assessed using the Shapiro–Wilk test and by examining normal probability plots.

As data for concurrent measurements of cPL concentrations and DGGR-lipase activities were not normally distributed, results are reported as median and interquartile range (IQR). Spearman’s rank (r_s_) was used to assess correlations between DGGR-lipase activity and cPL concentrations; for all correlation analyses, cases with cPL concentrations reported as greater than or less than the measurement range (most commonly <30 µg/L or >1500 µg/L) were excluded. Receiver operating characteristic (ROC) curves were used to assess the diagnostic accuracy of DGGR-lipase activity to predict cPL concentrations. Youden’s index (differential positive rate), calculated from the ROC curves, was used to determine optimal cutoffs for DGGR-lipase activity corresponding to cPL concentrations >200 µg/L and ≥400 µg/L, respectively. Sensitivities, specificities, positive and negative likelihood ratios, and predictive values were calculated for the optimal cutoff and for cutoffs corresponding to our URL (180 U/L) and 2× URL (360 U/L). Agreement between DGGR-lipase activity and cPL concentration was calculated using Cohen’s kappa coefficient (κ), whereby agreement was considered moderate (κ: 0.41–0.60), substantial (κ: 0.61–0.80), or near perfect (κ: 0.81–1.00). The ROC curves for predicting a clinical diagnosis of AP with both DGGR-lipase activity and cPL concentrations were used to assess the diagnostic accuracy of both tests, and the difference between the areas under the ROC curves (AUCs) were evaluated. Statistical significance was set at *p* ≤ 0.05 throughout.

## 3. Results

### 3.1. Animals

**Figure 1:**
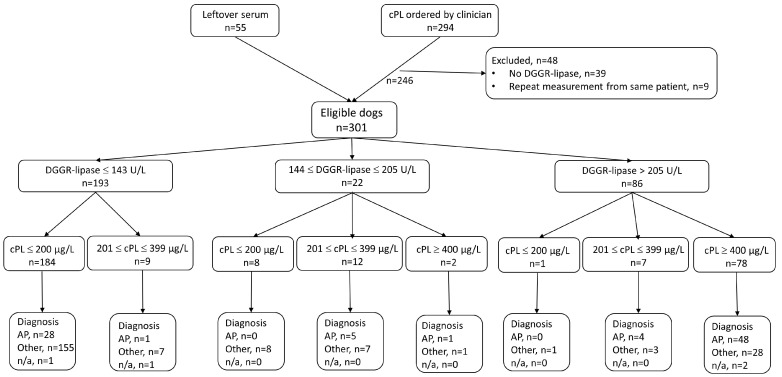
Flow chart depicting the selection of cases and summary of results. Abbreviations: DGGR: 1,2-o-dilauryl-rac-glycero-3-glutaric acid-(6’-methylresorufin) ester, cPL: canine pancreas-specific lipase, AP: acute pancreatitis, n/a: not available.

In total, 301 dogs met the inclusion criteria: 246 with cPL measurements requested by clinicians, and 55 for which cPL was measured from leftover serum (Figure 1). The dogs included 166 males (55%; 67 entire and 99 castrated) and 135 females (45%; 26 entire and 109 spayed) with ages ranging from 0.3–16.9 years (median 8.3 years, IQR 4.5–10.8). The study population was composed of 83 breeds, the most common being mixed breed (n = 58), Yorkshire Terrier (n = 17), Jack Russel Terrier (n = 14), French Bulldog (n = 13), Labrador Retriever (n = 11), and Chihuahua (n = 10), with less than 10 each for all other breeds.

### 3.2. Clinical Diagnoses

Eighty-seven of 301 (28.9%) cases had a clinical diagnosis of AP, 210 dogs (69.8%) had other diagnoses, and AP could neither be ruled in nor out in four dogs (1.3%) due to incomplete records; these cases were omitted from analysis involving clinical diagnosis. An abdominal ultrasound was performed in 248 dogs (82.4%), of which sonographic findings compatible with AP were reported in 55 (22.2%) cases. In dogs with disorders other than AP, gastrointestinal disorders were the most common (n = 111; 37.4%), followed by urogenital (n = 19, 6.4%), neoplastic (n = 18, 6%), neurologic (n = 15, 5%), endocrine (n = 12, 4%) and hepatobiliary (n = 10, 3.4%) disorders. Other organ systems were affected by six (2%) or fewer cases each. Three dogs (1%) had pancreatic disorders other than AP, including one adenocarcinoma, one exocrine pancreatic insufficiency, and one suspicion of nesidioblastosis. No final diagnosis could be reached for five dogs (1.7%) for which AP was clinically ruled out.

### 3.3. DGGR-Lipase Activities and cPL Concentrations

DGGR-lipase activities ranged from 10–15,616 U/L and cPL concentrations ranged from 8 to >2000 µg/L (Table 1). Both DGGR-lipase activities and cPL concentrations were significantly (*p* < 0.001) higher in dogs with a clinical diagnosis of AP than in dogs with other diagnoses (Table 1).

**Figure 2:**
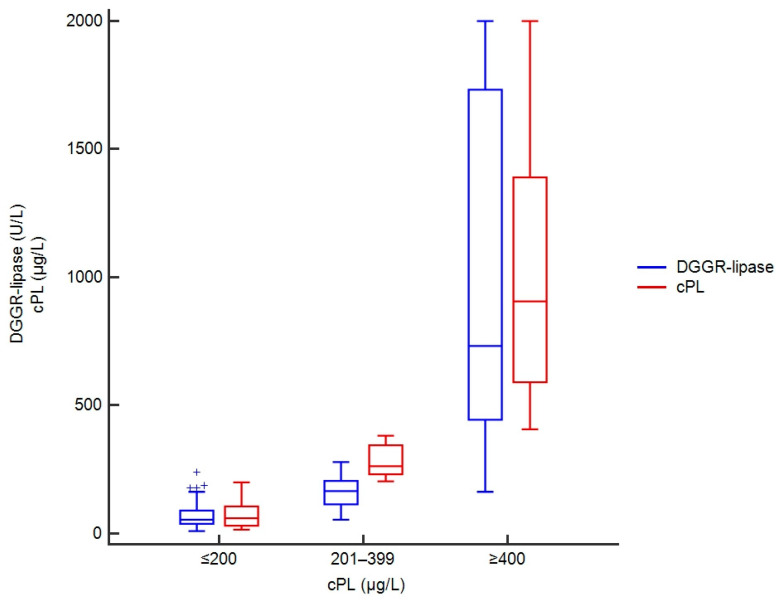
Box plots showing DGGR-lipase activities and cPL concentrations in 301 dogs, stratified by cPL categories. For cPL concentrations reported as greater than or less than a value, the absolute values were taken, and DGGR-lipase activity was capped at 2000 U/L to avoid stretching of the graph due to a few extremely high values.

DGGR-lipase activities ranged from 10–238 U/L in samples with concentrations of cPL ≤ 200 µg/L, 54–279 U/L in samples with cPL 201–399 µg/L, and 163–15,616 U/L in samples with cPL ≥ 400 µg/L (Figure 2).

**Figure 3:**
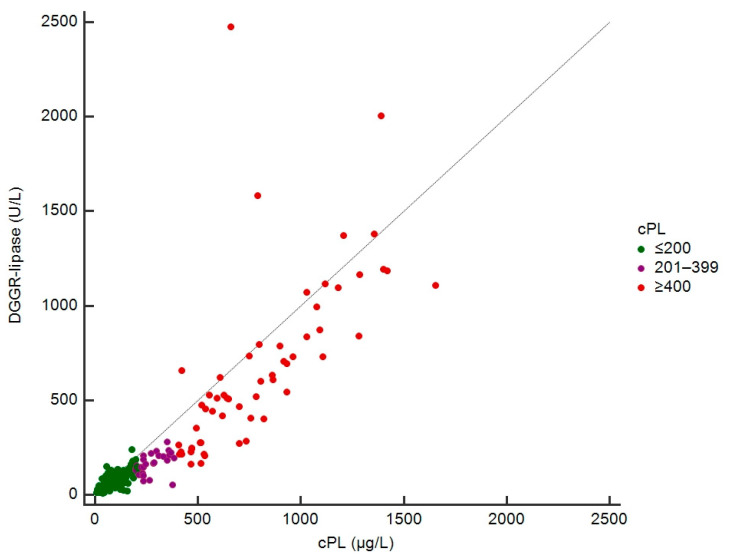
Scatterplot of DGGR-lipase and cPL concentrations in 247 dogs.

Absolute cPL concentrations were available in 247 dogs for correlation analyses with DGGR-lipase activities. The rank correlation (r_s_) was 0.91 (95% confidence interval (CI): 0.88–0.93; *p* < 0.001) (Figure 3).

### 3.4. Accuracy of DGGR-Lipase Activity to Predict cPL Concentrations

**Figure 4:**
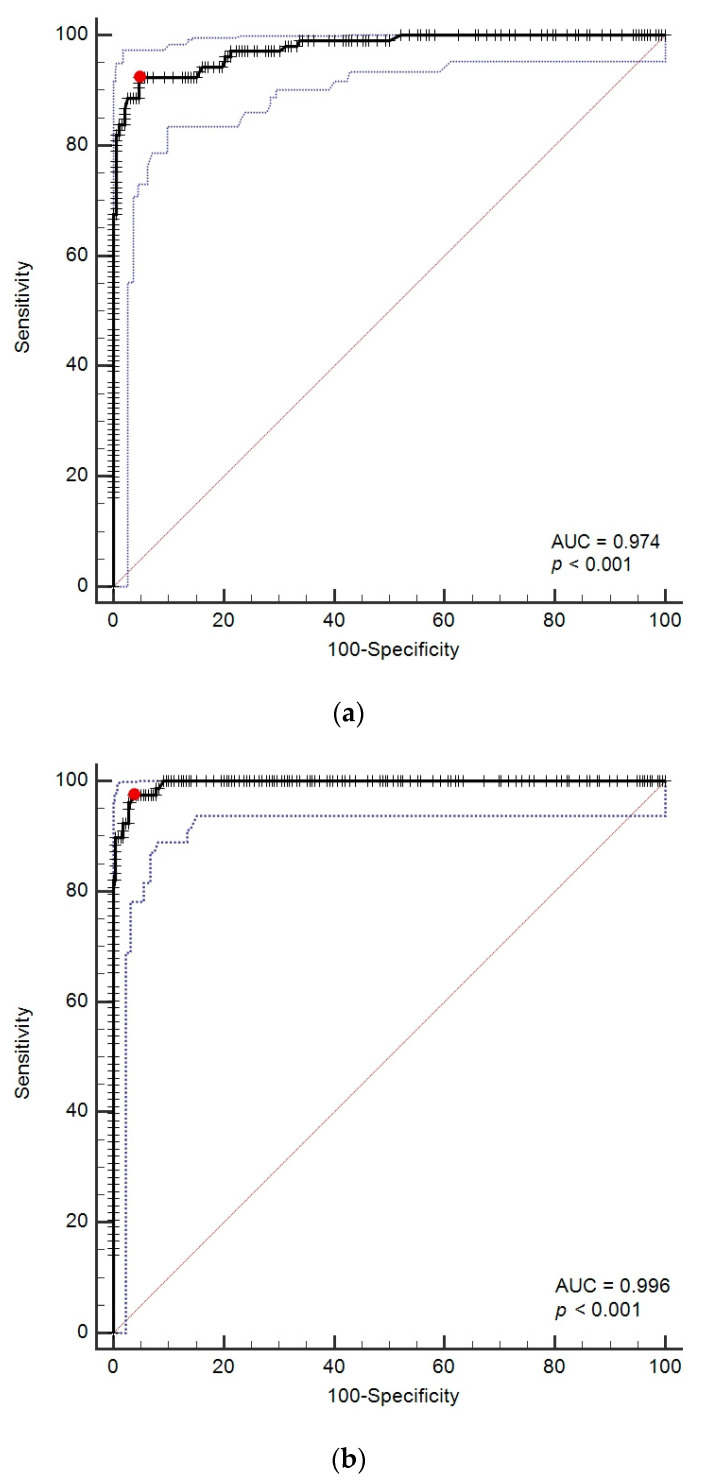
(**a**) Receiver operating characteristic (ROC) curves for prediction of cPL > 200 µg/L with DGGR-lipase. (**b**) ROC curves for prediction of cPL ≥ 400 µg/L with DGGR-lipase. The red dot represents the optimal cutoff calculated by Youden’s index; the blue dotted lines represent confidence intervals. Abbreviation: AUC: areas under the ROC curves.

The AUCs of DGGR-lipase activity to predict cPL concentrations >200 µg/L and ≥400 µg/L were 0.974 (95% CI, 0.949–0.989) and 0.996 (95% CI, 0.980–1.000), respectively (Figure 4).

Optimal cutoffs for DGGR-lipase activity were >143 U/L (95% CI, 136–188) and >205 U/L (95% CI, 146–213) for cPL concentrations >200 µg/L and ≥400 µg/L, respectively (Table 2 and Table 3). The agreement between DGGR-lipase activity and cPL concentrations for the optimal cutoffs and for cutoffs of 1× URL and 2× URL are summarized in Table 4.

There were 32 dogs with DGGR-lipase activity >205 U/L that had a diagnosis other than pancreatitis (Figure 1). Of these cases, 28 also had cPL concentrations ≥400 µg/L; the urogenital tract was the most commonly affected organ system (n = 9). Only one dog had a DGGR-lipase activity >205 U/L with a cPL concentration of <200 µg/L and was diagnosed with inflammatory bowel disease; the remaining three dogs had gastrointestinal disorders and cPL concentrations of 201–399 µg/L (equivocal range). There were 193 dogs with DGGR-lipase activity ≤143 U/L, of which 184 dogs also had cPL concentrations ≤200 µg/L and the remaining 9 dogs had concentrations of 201–399 µg/L (Figure 1).

### 3.5. Accuracy of DGGR-Lipase Activities and cPL Concentrations for a Clinical Diagnosis of Acute Pancreatitis

**Figure 5:**
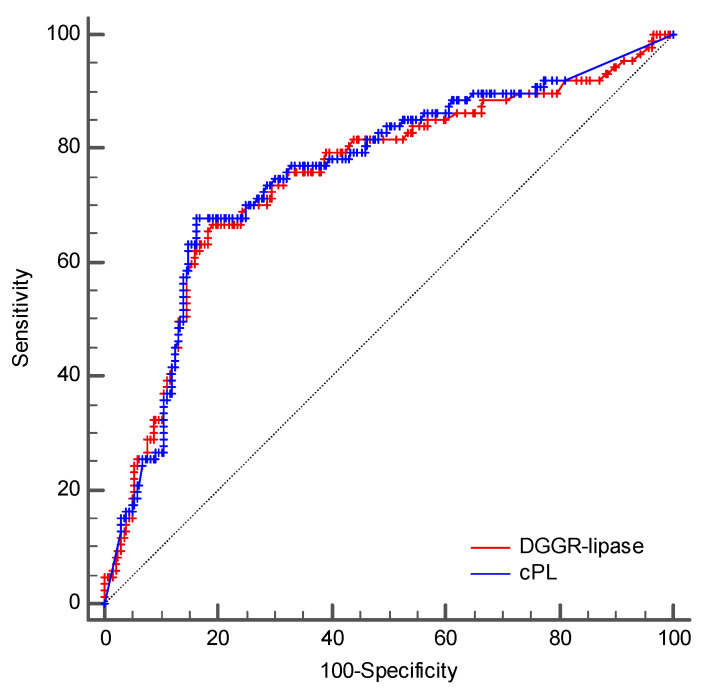
Comparison of the ROC curves of DGGR-lipase activity and cPL concentrations for a clinical diagnosis of AP.

The AUCs to predict a clinical diagnosis of AP were 0.748 (95% CI, 0.695–0.797) and 0.756 (95% CI, 0.703–0.803) for DGGR-lipase activity and cPL concentrations, respectively (Figure 5). The optimal cutoffs for a clinical diagnosis of AP were >161 U/L for DGGR-lipase activity and >235 µg/L for cPL concentrations, respectively. The accuracies of both assays for a clinical diagnosis of AP at different cutoffs are summarised in Table 5 and Table 6. The difference between the two AUCs was insignificant (difference in areas, 0.008; 95% CI, −0.014 to 0.029; *p* = 0.4778) (Figure 5).

## 4. Discussion

In this study, we evaluated DGGR-lipase activity and cPL concentrations in dogs with a variety of different disorders. Although most cPL measurements were requested by clinicians, presumably because pancreatitis was considered a differential diagnosis to be confirmed or ruled out, we also included leftover samples from dogs for which cPL was not initially requested in order to cover a wide range of clinical diseases.

The results of our study showed a very good correlation (r_s_ 0.91) between DGGR-lipase activity and cPL concentrations and are similar to findings of previous studies which demonstrated rank correlations of 0.90 and 0.93 [23,24]. We also demonstrated near perfect agreement (κ 0.87 and 0.92) between DGGR-lipase activity of >143 U/L and >205 U/L and cPL concentrations >200 and ≥400 µg/L, respectively. This was higher than previously reported agreements of 0.70–0.80 and 0.55–0.80 for cPL concentrations >200 and ≥400 µg/L, respectively, using cutoffs for DGGR-lipase of 1× URL, 1.5× URL, and 2× URL [23]. It was also much higher than another study reporting κ 0.70 for cPL concentrations ≥400 µg/L using a cutoff of 1× URL for DGGR-lipase [24]. However, the latter study was based on histologically confirmed pancreatic findings in 18 dogs, of which only 3 had AP and 8 had chronic pancreatitis [24]. The superior agreement found in our study is likely due, at least in part, to the calculation of optimal cutoffs, which substantially improved agreement, particularly for cPL concentrations ≥400 µg/L, compared to using 2× URL as the cutoff. However, a direct comparison between studies is difficult in part due to differences in the DGGR-lipase assays and analysers used, disease severities, and dog populations. Nonetheless, using optimal cutoffs for the prediction of cPL concentrations, we found DGGR-lipase activity to have very high accuracy with positive and negative predictive values of over 90%, as well as excellent likelihood ratios to predict cPL concentrations >200 and ≥400 µg/L. Given that the cPL assay is considered specific for pancreatic lipase, the strong agreement between DGGR-lipase activity and cPL concentrations suggest that extrapancreatic lipases do not substantially contribute to DGGR-lipase activity in most clinical cases. These findings suggest that if DGGR-lipase activity is measured, additional measurement of cPL concentrations in an external laboratory with higher costs does not add clinically useful information in the vast majority of cases. Indeed, based on the cutoffs we established for optimal agreement between the assays, we found only one dog with a DGGR-lipase activity >205 U/L that had a cPL concentration ≤200 μg/L, and no dog with DGGR-lipase activity ≤143 U/L had a cPL concentration ≥400 μg/L.

However, of dogs with a clinical diagnosis, we found 29/78 (37%) with cPL concentrations ≥400 µg/L that had a disorder other than AP, and 38/219 (17%) of dogs with cPL concentrations <400 µg/L were considered to have AP. Indeed, the optimal cutoffs we found associated with a clinical diagnosis of AP were in the equivocal diagnostic range for cPL concentrations (235 µg/L) and within the upper end (161 U/L) of our reference interval of 25–180 U/L for DGGR-lipase activity. Moreover, the diagnostic accuracy for both tests was not high, with sensitivities and specificities of 68% and 84%, and 67% and 81% for cPL concentrations and DGGR-lipase activity, respectively. Positive and negative likelihood ratios calculated for different DGGR-lipase and cPL cutoffs were also poor, suggesting that lipase measurement, regardless of the method used, is unable to reliably predict a clinical diagnosis of AP independently of the disease prevalence in the target population.

Our calculated sensitivities and specificities are lower than those reported by one previous study using a different DGGR-lipase assay (Precision pancreatic sensitive lipase (PSL), Antech Diagnostics) and a cutoff of >216 U/L, which showed sensitivities and specificities ranging between 81–91% and 74–81%, and 86–91% and 64–74% for cPL concentrations and DGGR-lipase activity, respectively [21]. However, in the latter study, only dogs with gastrointestinal disorders were included, a consensus score of a panel of internists was used for diagnosis, and only 12/50 dogs were considered to unequivocally have pancreatitis [21]. In a more recent study, a sensitivity and specificity of 81% and 82% was found for DGGR-lipase activity using an optimal cutoff based on ROC curve analysis to predict a clinical diagnosis of AP [25]. However, a clinical diagnosis of AP was largely based on either consistent ultrasonographic findings or cPL concentrations ≥400 µg/L, but 30% of dogs considered to have AP had cPL concentrations <400 µg/L [25]. Furthermore, the optimal cutoff calculated for DGGR-lipase (42.15 U/L) in that study was in the lower third of their reference interval, raising questions regarding the clinical utility of this cutoff [25]. Moreover, this cutoff was far lower than the optimal cutoff (161 U/L) for DGGR-lipase activity to predict a clinical diagnosis of AP found in the present study, suggesting a discrepancy between said previous study and ours regarding the criteria used to diagnose AP. Despite this, both studies found substantial numbers of dogs given a clinical diagnosis of AP with cPL concentrations ≤400 µg/L and/or DGGR-lipase activities within the reference interval [25]. One reason may be that ultrasonographic findings influenced the clinical diagnosis in many cases regardless of the results of either of the lipase assays. This assumption appears to be corroborated by previous studies demonstrating poor agreement between the assays and an ultrasonographic diagnosis of AP, prompting authors to urge caution when interpreting pancreatic ultrasound findings [23]. Another study showed either poor sensitivity or poor specificity of ultrasound for a diagnosis of AP depending on the sonographic criteria used, prompting authors to discourage the sole use of ultrasound to diagnose AP [4]. Furthermore, most studies evaluating the accuracy of cPL concentrations or DGGR-lipase activity for a clinical diagnosis of AP have not specifically evaluated the time point at which ultrasound was performed, although ultrasonographic findings influenced the diagnosis. As clinical signs and lipase assays may peak well before ultrasound findings consistent with AP are evident [27], both overdiagnosis of AP due to poor ultrasound specificity and underdiagnosis of AP due to early ultrasonographic examination or mild pancreatic changes are possible.

That many dogs in our study were not considered to have AP despite both high DGGR-lipase activity and cPL concentrations is unsurprising given that several previous studies have shown elevations in both assays in association with nonpancreatic disorders in the absence of clinical pancreatitis [13,14,15,16,17,18,19,20,26,28]. As histopathologies were not performed for the dogs in our study, subclinical or secondary pancreatitis may have been missed, leading potentially to an underestimation of the prevalence of AP. However, we found equal diagnostic accuracy for both assays for a clinical diagnosis of AP, suggesting that both methods are similarly influenced either by extrapancreatic disease and/or by diagnostic error, at least in the study population evaluated in this study.

The specificity of lipase activity to detect pancreatic inflammation has been contested in previous studies, most of which investigated 1,2-DiG [5,6], with only a few focusing on the DGGR-lipase assay. One study demonstrated a transient surge in DGGR-lipase, but not cPL, after injection of heparin in dogs, suggesting that the DGGR-lipase assay also detects hepatic and/or lipoprotein lipase [29]. It is, however, noteworthy that the peak DGGR-lipase activity, despite significantly differing from baseline, was minimal (median 54.1 U/L vs 49.8 U/L)—a degree which would not influence a clinical decision regarding a diagnosis of AP. Additionally, a conference abstract described residual DGGR-lipase activity in dogs with exocrine pancreatic insufficiency, suggesting that DGGR is not solely hydrolysed by pancreatic lipase [30]. However, the remaining lipase activity was mainly distributed in the lower half of the reference interval and therefore it is questionable how much extrapancreatic lipases can contribute to overall lipase activity as measured by the DGGR assay. If the cPL assay is as specific for pancreatic lipase as is claimed, then the high correlation and agreement between DGGR-lipase and cPL found in our study would suggest that diseases other than clinically overt pancreatitis can lead to significant increases in pancreatic lipase, with extrapancreatic lipases contributing minimally in most cases.

The present study has some important limitations, mainly owing to the retrospective study design, which carries a higher risk of bias arising from inaccurate or incomplete data. A clinical diagnosis of AP was not based on specific standardized criteria nor on a consensus panel of board-certified internal medicine specialists and histopathologic confirmation was not available. Given the near perfect agreement and excellent correlation between DGGR-lipase and cPL assays in our study, we can conclude that both measurements were influenced equally by these shortcomings in diagnosis; even if some cases would have been classified differently had histopathology been available, this would have shifted the diagnostic accuracy of both tests nearly equally. In addition, most cases in our institution are referred from primary practice, and most samples were therefore not taken at the initial onset of clinical signs. Whether or not a similarly high agreement between the assays would be found in the peracute phase of AP is unclear. Furthermore, our study design did not allow us to speculate as to the severity of AP, and agreement between the assays as well as diagnostic accuracy are likely highly dependent on whether mild or severe cases of AP are investigated. Thus, our findings may only be applicable to similar clinical settings and populations. Moreover, the specific cutoffs found in the present study cannot be assumed to apply when using other DGGR-lipase assays.

## 5. Conclusions

In conclusion, the results of this study suggest that DGGR-lipase activity is a highly accurate predictor of cPL concentrations in dogs, and that both assays have comparably modest accuracy to diagnose pancreatitis. The main advantages of measuring DGGR-lipase activity over cPL concentrations are availability and price. As DGGR-lipase activity is routinely run in our diagnostic laboratory, the results are available in under an hour at a cost to the client of around one-tenth of that of cPL concentrations, the results of which are received the following day. Whilst we acknowledge that only selected institutions have quick access to a diagnostic laboratory with an automated wet chemistry analyser, the low cost allows for DGGR-lipase activity to be used for monitoring during hospitalization, whereas cPL concentrations are rarely requested by clinicians in our institution more than once during a single episode of clinical disease.

## Figures and Tables

**Table 1:** Summary of DGGR-lipase and cPL values for all dogs, stratified by clinical diagnosis.

Variable	All Dogs (n = 301)	Clinical Diagnosis (n = 297)	
		Acute Pancreatitis (n = 87)	Other (n = 210)
DGGR-lipase (U/L), range (median; IQR)	10–15, 616 (91; 46–233)	19–15,616 (234; 111–827)	10–3592 (70; 42–132)
cPL (µg/L), range (median; IQR)	8.1–>2000 (111; 45–433)	<15–>2000 (464; 150–936)	8–>1500 (79; 37–188)
cPL ≤ 200, n (%)	193 (64.1%)	28 (14.6%)	164 (85.4%)
cPL 201–399, n (%)	28 (9.3%)	10 (37.0%)	17 (63.0%)
cPL ≥ 400, n (%)	80 (26.6%)	49 (62.8%)	29 (37.2%)

**Table 2:** Accuracy of DGGR-lipase activity (U/L) to predict cPL concentrations >200 µg/L in 301 dogs using the optimal cutoff based on Youden’s index and cutoffs corresponding to URL.

DGGR- Lipase Cutoff (U/L)	cPL (µg/L)		Total (n)	Sensitivity (95% CI)	Specificity (95% CI)	PPV (95% CI)	NPV (95% CI)	PLR (95% CI)	NLR (95% CI)	Accuracy (95% CI)
	≤200 (n)	>200 (n)								
≤143	184	9	193	92% (85–96%)	95% (91–98%)	92% (85–95%)	95% (92–97%)	19.7 (10.4–37.3)	0.1 (0.0–0.1)	94% (91–96%)
>143 (optimal)	9	99	108							
≤180	191	18	209	83% (75–90%)	99% (96–100%)	98% (92–99%)	91% (90–96%)	80.4 (20.2–320.1)	0.2 (0.2–0.3)	93% (90–96%)
>180 (>URL)	2	90	92							

CI: confidence intervals, PPV: positive predictive value, NPV: negative predictive value, PLR: positive likelihood ratio, NLR: negative likelihood ratio, URL: upper reference limit.

**Table 3:** Accuracy of DGGR-lipase activity (U/L) to predict cPL concentrations ≥400 µg/L in 301 dogs using the optimal cutoff based on Youden’s index and cutoffs corresponding to URL and 2× URL.

DGGR- Lipase Cutoff (U/L)	cPL (µg/L)		Total (n)	Sensitivity (95% CI)	Specificity (95% CI)	PPV (95% CI)	NPV (95% CI)	PLR (95% CI)	NLR (95% CI)	Accuracy (95% CI)
	<400 (n)	≥400 (n)								
<180	14	78	92	98% (91–100%)	94% (90–96%)	85% (77–90%)	99% (96–100%)	15.4 (9.2–25.6)	0.0 (0.0–0.1)	95% (92–97%)
>180 (>URL)	207	2	209							
≤205	213	2	215	98% (93–100%)	96% (93–98%)	91% (83–95%)	99% (96–100%)	26.9 (13.6–53.2)	0.0 (0.0–0.1)	97% (94–98%)
>205 (optimal)	8	78	86							
≤360	221	17	238	79% (68–87%)	100% (98–100%)	100%	93% (90–95%)	–	0.2 (0.1–0.4)	94% (91–97%)
>360 (>2× URL)	0	63	63							

**Table 4:** Agreement between DGGR lipase activity and cPL concentrations based on Youden’s index-derived optimal cutoffs for DGGR-lipase and cutoffs of URL and 2× URL.

DGGR-Lipase Cutoff (U/L)	cPL > 200 µg/L	cPL ≥ 400 µg/L
>143 U/L (optimal)	0.87 (0.81–0.93)	
>180 U/L (URL)	0.85 (0.79–0.91)	
>205 U/L (optimal)		0.92 (0.87–0.97)
>360 U/L (2× URL)		0.85 (0.77–0.92)

**Table 5:** Diagnostic accuracy of DGGR-lipase activities for a clinical diagnosis of acute pancreatitis in 297 dogs using the optimal cutoff based on Youden’s index, optimal cutoffs to predict cPL concentrations of >200 µg/L and ≥400 µg/L, and cutoffs corresponding to URL and 2× URL.

DGGR- Lipase Cutoff (U/L)	AP (n)	Other (n)	Total (n)	Sensitivity (95% CI)	Specificity (95% CI)	PPV (95% CI)	NPV (95% CI)	PLR (95% CI)	NLR (95% CI)	Accuracy (95%CI)
≤143	29	162	191	67% (56–76%)	77% (71–83%)	55% (48–62%)	85% (80–88%)	2.9 (2.2–3.9)	0.4 (0.3–0.6)	74% (69–79%)
>143	58	48	106							
≤161	29	170	199	67% (56–76%)	81% (75–86%)	59% (51–67%)	85% (81–89%)	3.5 (2.6–4.8)	0.4 (0.3–0.6)	77% (72–81%)
>161 (optimal)	58	40	98							
≤205	35	178	213	60% (49–70%)	85% (79–89%)	62% (53–70%)	84% (80–87%)	3.9 (2.7–5.6)	0.5 (0.4–0.6)	77% (72–82%)
>205	52	32	84							
≤180	32	175	207	63% (52–73%)	83% (78–88%)	61% (53–69%)	85% (80–88%)	3.8 (2.7–5.3)	0.4 (0.3–0.6)	77% (72–82%)
>180 (URL)	55	35	90							
≤360	51	184	235	41% (31–52%)	88% (83–92%)	58% (47–67%)	78% (75–81%)	3.3 (2.2–5.2)	0.7 (0.6–0.8)	74% (69–79%)
>360 (2× URL)	36	26	62							

**Table 6:** Diagnostic accuracy of cPL concentrations for a clinical diagnosis of acute pancreatitis in 297 dogs using the optimal cutoff based on Youden’s index and cutoffs considered equivocal (>200 µg/L) and consistent with pancreatitis (≥400 µg/L).

cPL Cutoff (µg/L)	AP (n)	Other (n)	Total (n)	Sensitivity (95% CI)	Specificity (95% CI)	PPV (95% CI)	NPV (95% CI)	PLR (95% CI)	NLR (95% CI)	Accuracy (95%CI)
≤200	164	28	192	68% (57–77%)	78% (72–83%)	56% (49–63%)	85% (81–89%)	3.1 (2.3–4.2)	0.4 (0.3–0.6)	75% (70–80%)
>200	59	46	105							
≤235	28	176	204	68% (57–77%)	84% (78–89%)	63% (55–71%)	86% (82–90%)	4.2 (3.0–5.9)	0.4 (0.3–0.5)	79% (74–84%)
>235 (optimal)	59	34	93							
<400	38	181	219	56% (45–67%)	86% (81–91%)	63% (53–71%)	83% (79–86%)	4.1 (2.8–6)	0.5 (0.4–0.6)	77% (72–82%)
≥400	49	29	78							

## Data Availability

The data presented in this study are openly available in FigShare at doi:10.6084/m9.figshare.19361624.
